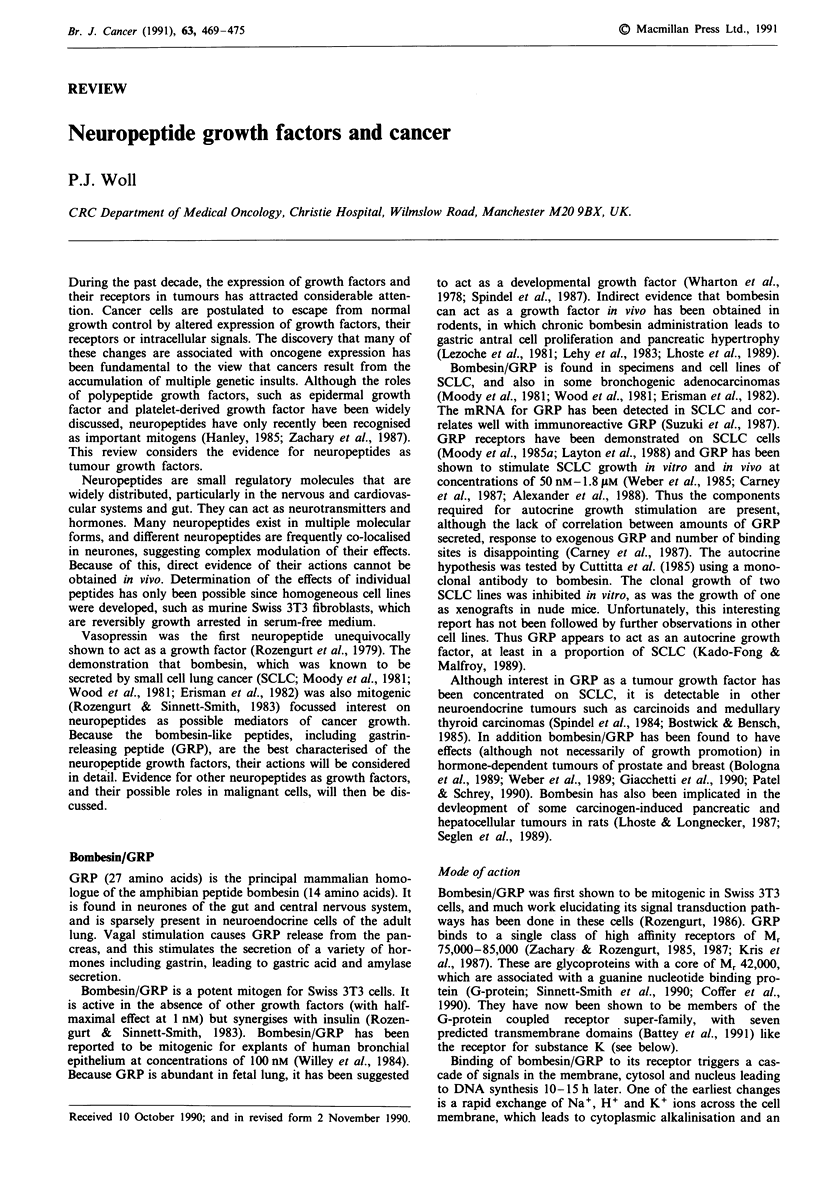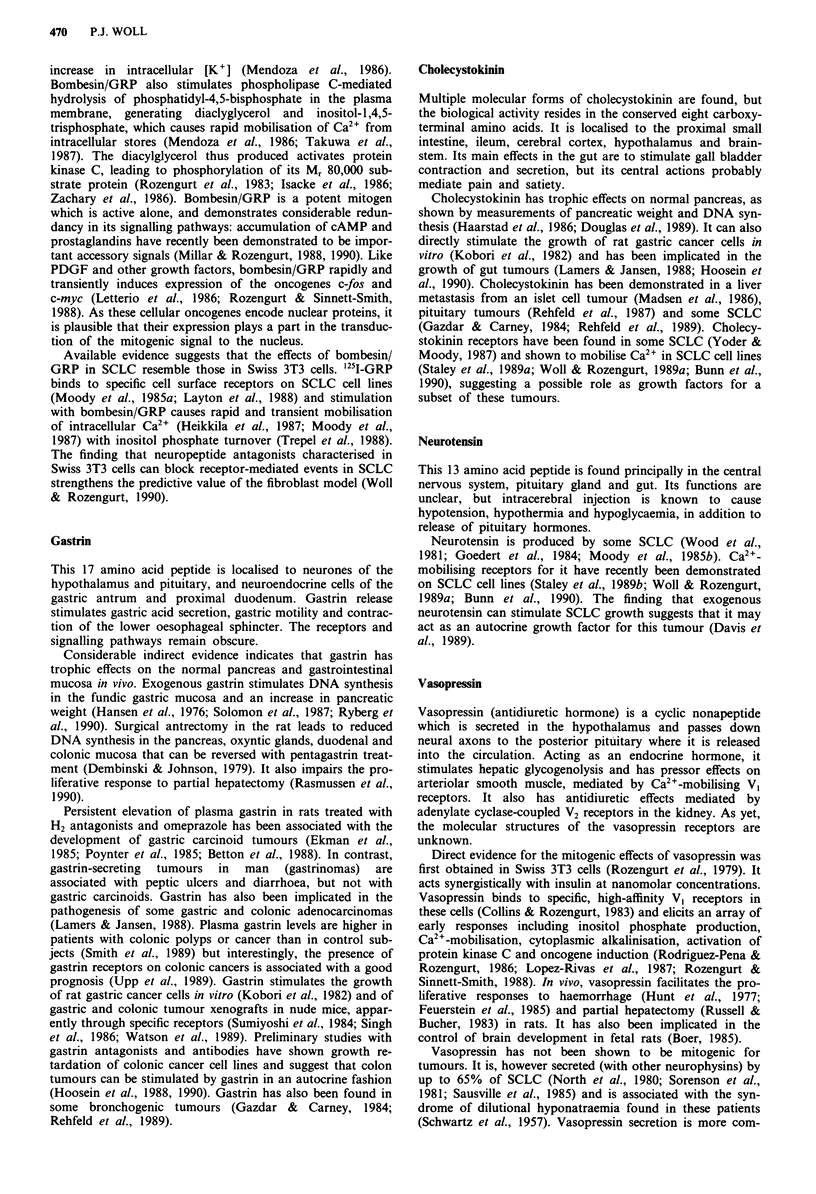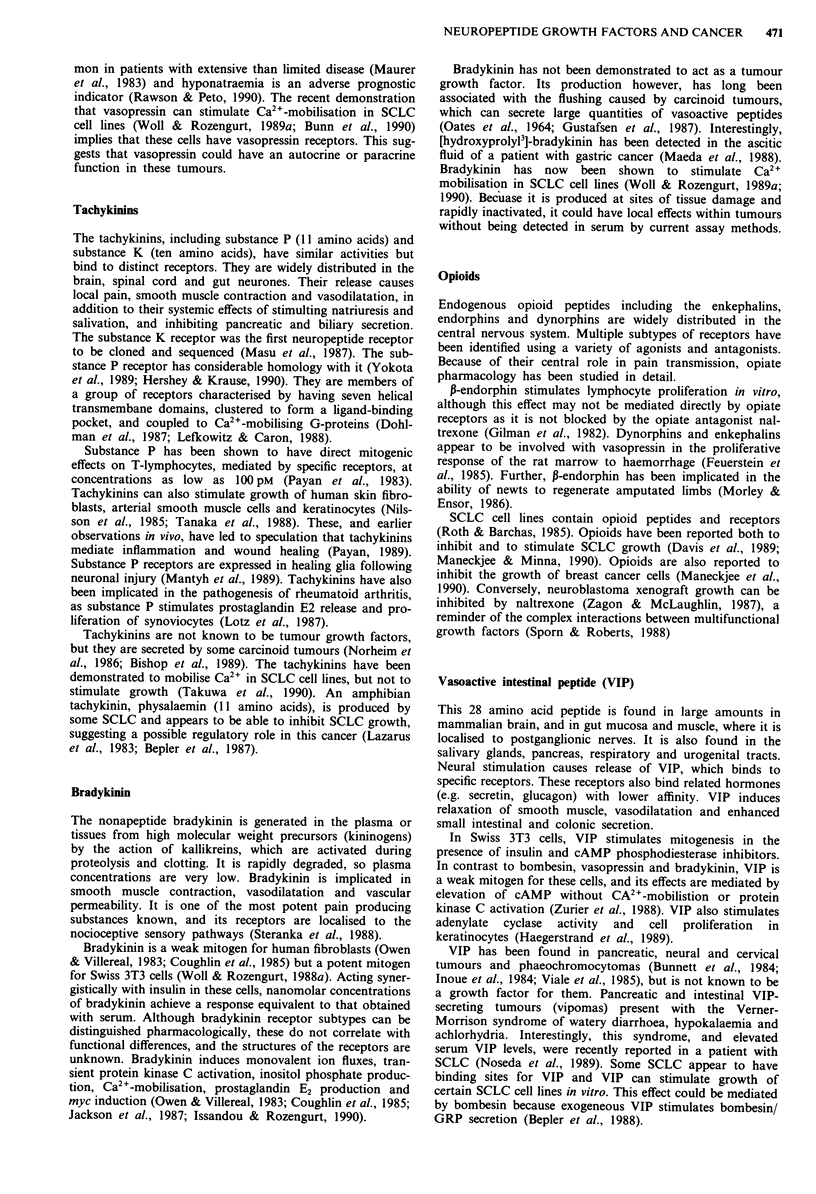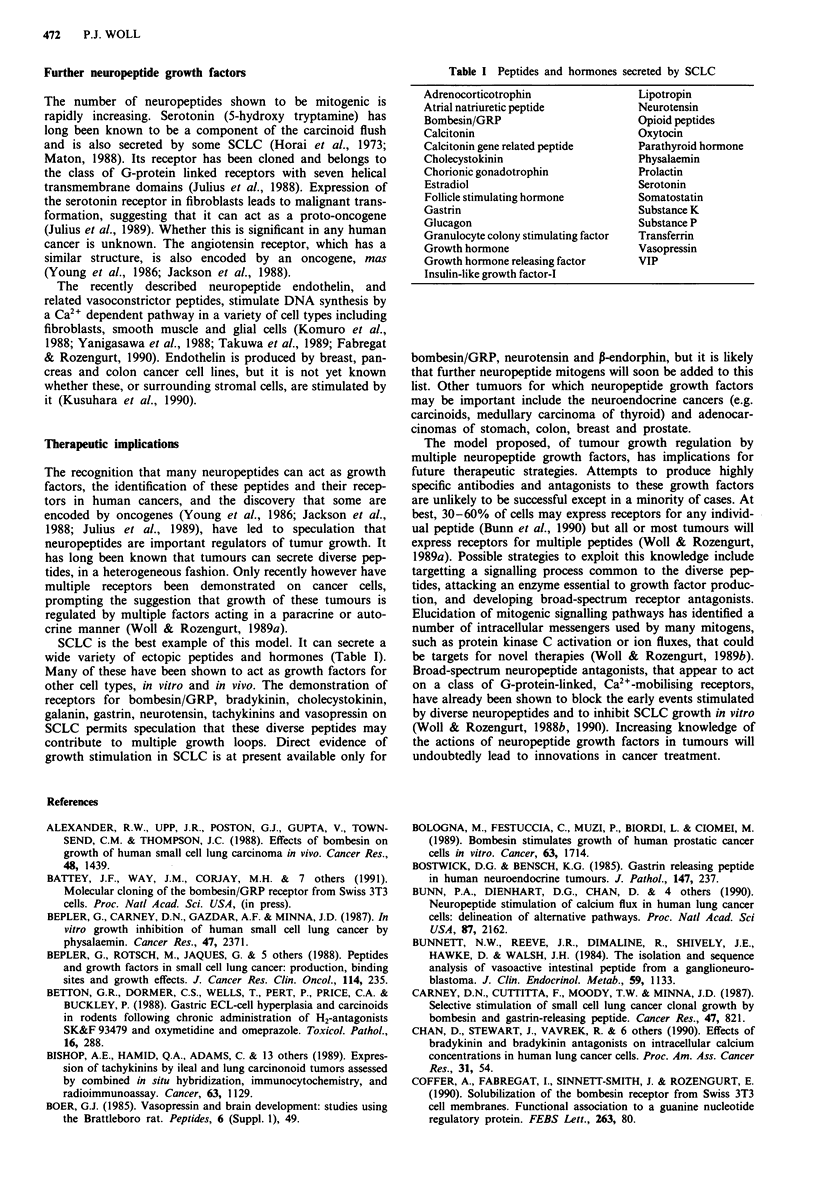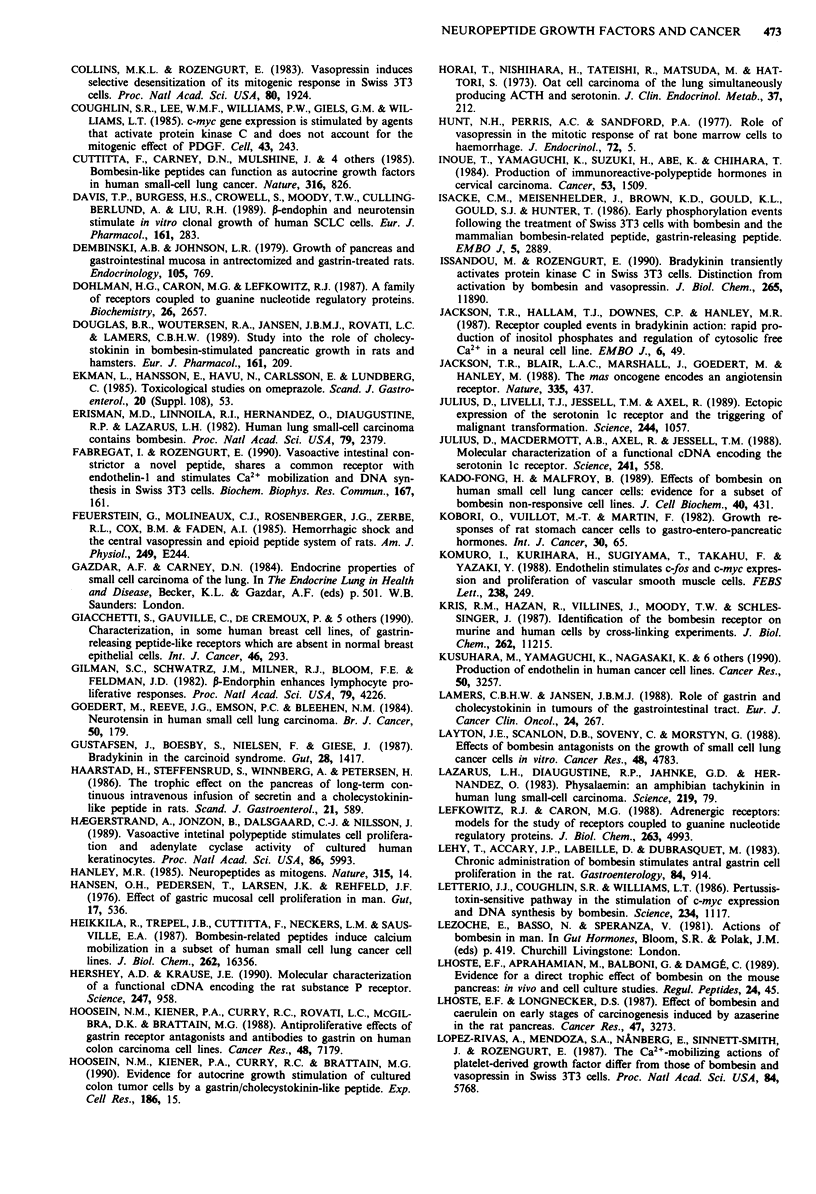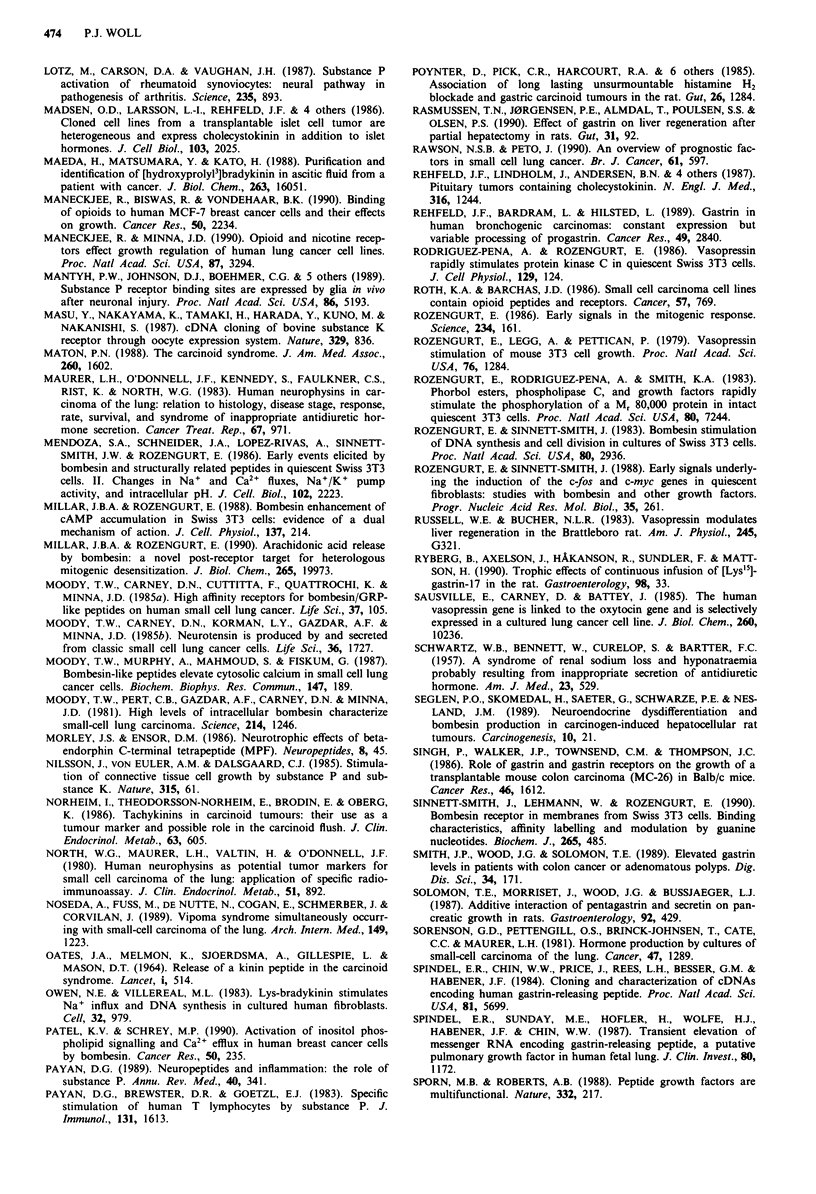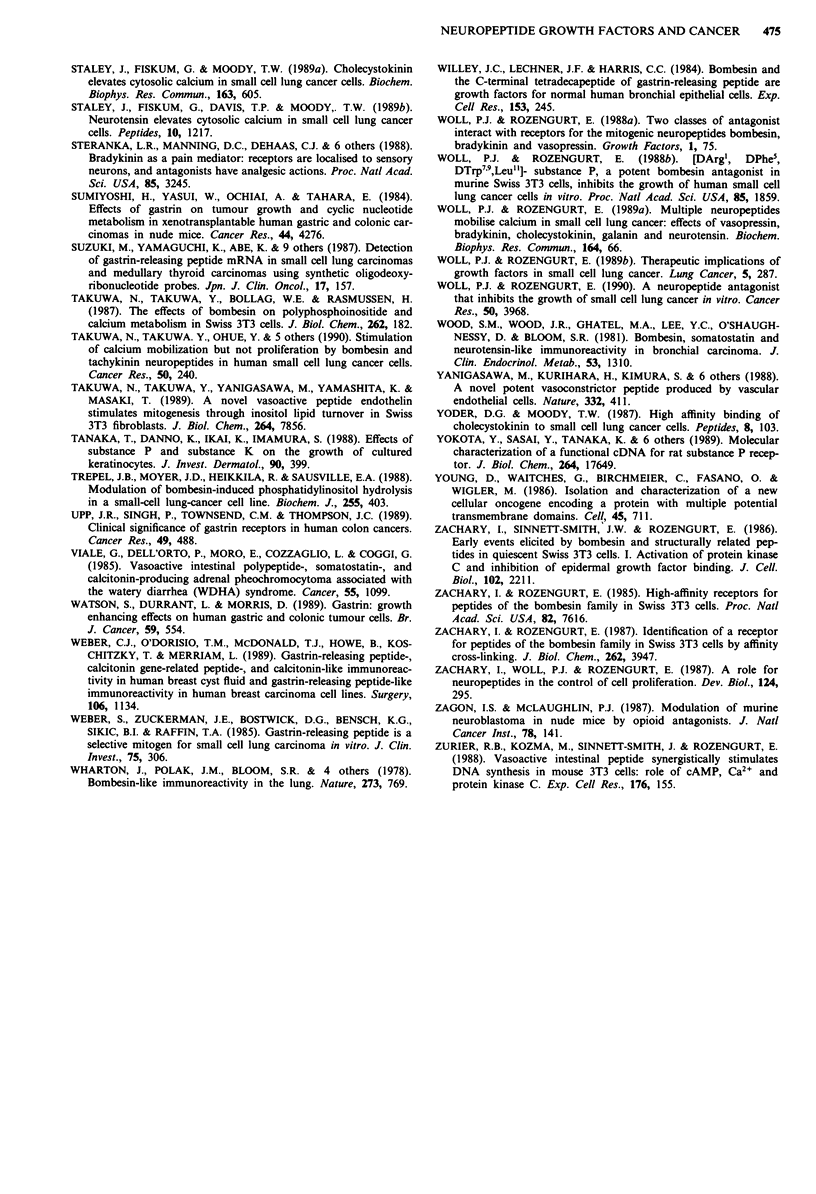# Neuropeptide growth factors and cancer.

**DOI:** 10.1038/bjc.1991.110

**Published:** 1991-03

**Authors:** P. J. Woll

**Affiliations:** CRC Department of Medical Oncology, Christie Hospital, Manchester, UK.


Br. J. Cancer (1991), 63, 469–475 © Macmillan Press Ltd., 1991

REVIEW

### Neuropeptide growth factors and cancer

P.J. Woll

*CRC Department of Medical Oncology, Christie Hospital, Wilmslow Road, Manchester M20 9BX, UK.*

During the past decade, the expression of growth factors and their receptors in tumours has attracted considerable attention. Cancer cells are postulated to escape from normal growth control by altered expression of growth factors, their receptors or intracellular signals. The discovery that many of these changes are associated with oncogene expression has been fundamental to the view that cancers result from the accumulation of multiple genetic insults. Although the roles of polypeptide growth factors, such as epidermal growth factor and platelet-derived growth factor have been widely discussed, neuropeptides have only recently been recognised as important mitogens (Hanley, 1985; Zachary *et al.*, 1987). This review considers the evidence for neuropeptides as tumour growth factors.

Neuropeptides are small regulatory molecules that are widely distributed, particularly in the nervous and cardiovascular systems and gut. They can act as neurotransmitters and hormones. Many neuropeptides exist in multiple molecular forms, and different neuropeptides are frequently co-localised in neurones, suggesting complex modulation of their effects. Because of this, direct evidence of their actions cannot be obtained *in vivo*. Determination of the effects of individual peptides has only been possible since homogeneous cell lines were developed, such as murine Swiss 3T3 fibroblasts, which are reversibly growth arrested in serum-free medium.

Vasopressin was the first neuropeptide unequivocally shown to act as a growth factor (Rozengurt *et al.*, 1979). The demonstration that bombesin, which was known to be secreted by small cell lung cancer (SCLC; Moody *et al.*, 1981; Wood *et al.*, 1981; Erisman *et al.*, 1982) was also mitogenic (Rozengurt & Sinnett-Smith, 1983) focussed interest on neuropeptides as possible mediators of cancer growth. Because the bombesin-like peptides, including gastrin-releasing peptide (GRP), are the best characterised of the neuropeptide growth factors, their actions will be considered in detail. Evidence for other neuropeptides as growth factors, and their possible roles in malignant cells, will then be discussed.

### Bombesin/GRP

GRP (27 amino acids) is the principal mammalian homologue of the amphibian peptide bombesin (14 amino acids). It is found in neurones of the gut and central nervous system, and is sparsely present in neuroendocrine cells of the adult lung. Vagal stimulation causes GRP release from the pancreas, and this stimulates the secretion of a variety of hormones including gastrin, leading to gastric acid and amylase secretion.

Bombesin/GRP is a potent mitogen for Swiss 3T3 cells. It is active in the absence of other growth factors (with half-maximal effect at 1 nM) but synergises with insulin (Rozengurt & Sinnett-Smith, 1983). Bombesin/GRP has been reported to be mitogenic for explants of human bronchial epithelium at concentrations of 100 nM (Willey *et al.*, 1984). Because GRP is abundant in fetal lung, it has been suggested to act as a developmental growth factor (Wharton *et al.*, 1978; Spindel *et al.*, 1987). Indirect evidence that bombesin can act as a growth factor *in vivo* has been obtained in rodents, in which chronic bombesin administration leads to gastric antral cell proliferation and pancreatic hypertrophy (Lezoche *et al.*, 1981; Lehy *et al.*, 1983; Lhoste *et al.*, 1989).

Bombesin/GRP is found in specimens and cell lines of SCLC, and also in some bronchogenic adenocarcinomas (Moody *et al.*, 1981; Wood *et al.*, 1981; Erisman *et al.*, 1982). The mRNA for GRP has been detected in SCLC and correlates well with immunoreactive GRP (Suzuki *et al.*, 1987). GRP receptors have been demonstrated on SCLC cells (Moody *et al.*, 1985*a*; Layton *et al.*, 1988) and GRP has been shown to stimulate SCLC growth *in vitro* and *in vivo* at concentrations of 50 nM–1.8 μM (Weber *et al.*, 1985; Carney *et al.*, 1987; Alexander *et al.*, 1988). Thus the components required for autocrine growth stimulation are present, although the lack of correlation between amounts of GRP secreted, response to exogenous GRP and number of binding sites is disappointing (Carney *et al.*, 1987). The autocrine hypothesis was tested by Cuttitta *et al.* (1985) using a monoclonal antibody to bombesin. The clonal growth of two SCLC lines was inhibited *in vitro*, as was the growth of one as xenografts in nude mice. Unfortunately, this interesting report has not been followed by further observations in other cell lines. Thus GRP appears to act as an autocrine growth factor, at least in a proportion of SCLC (Kado-Fong & Malfroy, 1989).

Although interest in GRP as a tumour growth factor has been concentrated on SCLC, it is detectable in other neuroendocrine tumours such as carcinoids and medullary thyroid carcinomas (Spindel *et al.*, 1984; Bostwick & Bensch, 1985). In addition bombesin/GRP has been found to have effects (although not necessarily of growth promotion) in hormone-dependent tumours of prostate and breast (Bologna *et al.*, 1989; Weber *et al.*, 1989; Giacchetti *et al.*, 1990; Patel & Schrey, 1990). Bombesin has also been implicated in the devleopment of some carcinogen-induced pancreatic and hepatocellular tumours in rats (Lhoste & Longnecker, 1987; Seglen *et al.*, 1989).

### *Mode of action*

Bombesin/GRP was first shown to be mitogenic in Swiss 3T3 cells, and much work elucidating its signal transduction pathways has been done in these cells (Rozengurt, 1986). GRP binds to a single class of high affinity receptors of $M_r$ 75,000–85,000 (Zachary & Rozengurt, 1985, 1987; Kris *et al.*, 1987). These are glycoproteins with a core of $M_r$ 42,000, which are associated with a guanine nucleotide binding protein (G-protein; Sinnett-Smith *et al.*, 1990; Coffer *et al.*, 1990). They have now been shown to be members of the G-protein coupled receptor super-family, with seven predicted transmembrane domains (Battey *et al.*, 1991) like the receptor for substance K (see below).

Binding of bombesin/GRP to its receptor triggers a cascade of signals in the membrane, cytosol and nucleus leading to DNA synthesis 10–15 h later. One of the earliest changes is a rapid exchange of $Na^+$, $H^+$ and $K^+$ ions across the cell membrane, which leads to cytoplasmic alkalinisation and an

Received 10 October 1990; and in revised form 2 November 1990.

increase in intracellular [$K^+$] (Mendoza *et al.*, 1986). Bombesin/GRP also stimulates phospholipase C-mediated hydrolysis of phosphatidyl-4,5-bisphosphate in the plasma membrane, generating diaclyglycerol and inositol-1,4,5-trisphosphate, which causes rapid mobilisation of $Ca^{2+}$ from intracellular stores (Mendoza *et al.*, 1986; Takuwa *et al.*, 1987). The diacylglycerol thus produced activates protein kinase C, leading to phosphorylation of its $M_r$ 80,000 substrate protein (Rozengurt *et al.*, 1983; Isacke *et al.*, 1986; Zachary *et al.*, 1986). Bombesin/GRP is a potent mitogen which is active alone, and demonstrates considerable redundancy in its signalling pathways: accumulation of cAMP and prostaglandins have recently been demonstrated to be important accessory signals (Millar & Rozengurt, 1988, 1990). Like PDGF and other growth factors, bombesin/GRP rapidly and transiently induces expression of the oncogenes c-*fos* and c-*myc* (Letterio *et al.*, 1986; Rozengurt & Sinnett-Smith, 1988). As these cellular oncogenes encode nuclear proteins, it is plausible that their expression plays a part in the transduction of the mitogenic signal to the nucleus.

Available evidence suggests that the effects of bombesin/GRP in SCLC resemble those in Swiss 3T3 cells. $^{125}$I-GRP binds to specific cell surface receptors on SCLC cell lines (Moody *et al.*, 1985*a*; Layton *et al.*, 1988) and stimulation with bombesin/GRP causes rapid and transient mobilisation of intracellular $Ca^{2+}$ (Heikkila *et al.*, 1987; Moody *et al.*, 1987) with inositol phosphate turnover (Trepel *et al.*, 1988). The finding that neuropeptide antagonists characterised in Swiss 3T3 cells can block receptor-mediated events in SCLC strengthens the predictive value of the fibroblast model (Woll & Rozengurt, 1990).

### Gastrin

This 17 amino acid peptide is localised to neurones of the hypothalamus and pituitary, and neuroendocrine cells of the gastric antrum and proximal duodenum. Gastrin release stimulates gastric acid secretion, gastric motility and contraction of the lower oesophageal sphincter. The receptors and signalling pathways remain obscure.

Considerable indirect evidence indicates that gastrin has trophic effects on the normal pancreas and gastrointestinal mucosa *in vivo*. Exogenous gastrin stimulates DNA synthesis in the fundic gastric mucosa and an increase in pancreatic weight (Hansen *et al.*, 1976; Solomon *et al.*, 1987; Ryberg *et al.*, 1990). Surgical antrectomy in the rat leads to reduced DNA synthesis in the pancreas, oxyntic glands, duodenal and colonic mucosa that can be reversed with pentagastrin treatment (Dembinski & Johnson, 1979). It also impairs the proliferative response to partial hepatectomy (Rasmussen *et al.*, 1990).

Persistent elevation of plasma gastrin in rats treated with $H_2$ antagonists and omeprazole has been associated with the development of gastric carcinoid tumours (Ekman *et al.*, 1985; Poynter *et al.*, 1985; Betton *et al.*, 1988). In contrast, gastrin-secreting tumours in man (gastrinomas) are associated with peptic ulcers and diarrhoea, but not with gastric carcinoids. Gastrin has also been implicated in the pathogenesis of some gastric and colonic adenocarcinomas (Lamers & Jansen, 1988). Plasma gastrin levels are higher in patients with colonic polyps or cancer than in control subjects (Smith *et al.*, 1989) but interestingly, the presence of gastrin receptors on colonic cancers is associated with a good prognosis (Upp *et al.*, 1989). Gastrin stimulates the growth of rat gastric cancer cells *in vitro* (Kobori *et al.*, 1982) and of gastric and colonic tumour xenografts in nude mice, apparently through specific receptors (Sumiyoshi *et al.*, 1984; Singh *et al.*, 1986; Watson *et al.*, 1989). Preliminary studies with gastrin antagonists and antibodies have shown growth retardation of colonic cancer cell lines and suggest that colon tumours can be stimulated by gastrin in an autocrine fashion (Hoosein *et al.*, 1988, 1990). Gastrin has also been found in some bronchogenic tumours (Gazdar & Carney, 1984; Rehfeld *et al.*, 1989).

### Cholecystokinin

Multiple molecular forms of cholecystokinin are found, but the biological activity resides in the conserved eight carboxy-terminal amino acids. It is localised to the proximal small intestine, ileum, cerebral cortex, hypothalamus and brainstem. Its main effects in the gut are to stimulate gall bladder contraction and secretion, but its central actions probably mediate pain and satiety.

Cholecystokinin has trophic effects on normal pancreas, as shown by measurements of pancreatic weight and DNA synthesis (Haarstad *et al.*, 1986; Douglas *et al.*, 1989). It can also directly stimulate the growth of rat gastric cancer cells *in vitro* (Kobori *et al.*, 1982) and has been implicated in the growth of gut tumours (Lamers & Jansen, 1988; Hoosein *et al.*, 1990). Cholecystokinin has been demonstrated in a liver metastasis from an islet cell tumour (Madsen *et al.*, 1986), pituitary tumours (Rehfeld *et al.*, 1987) and some SCLC (Gazdar & Carney, 1984; Rehfeld *et al.*, 1989). Cholecystokinin receptors have been found in some SCLC (Yoder & Moody, 1987) and shown to mobilise $Ca^{2+}$ in SCLC cell lines (Staley *et al.*, 1989*a*; Woll & Rozengurt, 1989*a*; Bunn *et al.*, 1990), suggesting a possible role as growth factors for a subset of these tumours.

### Neurotensin

This 13 amino acid peptide is found principally in the central nervous system, pituitary gland and gut. Its functions are unclear, but intracerebral injection is known to cause hypotension, hypothermia and hypoglycaemia, in addition to release of pituitary hormones.

Neurotensin is produced by some SCLC (Wood *et al.*, 1981; Goedert *et al.*, 1984; Moody *et al.*, 1985*b*). $Ca^{2+}$-mobilising receptors for it have recently been demonstrated on SCLC cell lines (Staley *et al.*, 1989*b*; Woll & Rozengurt, 1989*a*; Bunn *et al.*, 1990). The finding that exogenous neurotensin can stimulate SCLC growth suggests that it may act as an autocrine growth factor for this tumour (Davis *et al.*, 1989).

### Vasopressin

Vasopressin (antidiuretic hormone) is a cyclic nonapeptide which is secreted in the hypothalamus and passes down neural axons to the posterior pituitary where it is released into the circulation. Acting as an endocrine hormone, it stimulates hepatic glycogenolysis and has pressor effects on arteriolar smooth muscle, mediated by $Ca^{2+}$-mobilising $V_1$ receptors. It also has antidiuretic effects mediated by adenylate cyclase-coupled $V_2$ receptors in the kidney. As yet, the molecular structures of the vasopressin receptors are unknown.

Direct evidence for the mitogenic effects of vasopressin was first obtained in Swiss 3T3 cells (Rozengurt *et al.*, 1979). It acts synergistically with insulin at nanomolar concentrations. Vasopressin binds to specific, high-affinity $V_1$ receptors in these cells (Collins & Rozengurt, 1983) and elicits an array of early responses including inositol phosphate production, $Ca^{2+}$-mobilisation, cytoplasmic alkalinisation, activation of protein kinase C and oncogene induction (Rodriguez-Pena & Rozengurt, 1986; Lopez-Rivas *et al.*, 1987; Rozengurt & Sinnett-Smith, 1988). *In vivo*, vasopressin facilitates the proliferative responses to haemorrhage (Hunt *et al.*, 1977; Feuerstein *et al.*, 1985) and partial hepatectomy (Russell & Bucher, 1983) in rats. It has also been implicated in the control of brain development in fetal rats (Boer, 1985).

Vasopressin has not been shown to be mitogenic for tumours. It is, however secreted (with other neurophysins) by up to 65% of SCLC (North *et al.*, 1980; Sorenson *et al.*, 1981; Sausville *et al.*, 1985) and is associated with the syndrome of dilutional hyponatraemia found in these patients (Schwartz *et al.*, 1957). Vasopressin secretion is more common in patients with extensive than limited disease (Maurer *et al.*, 1983) and hyponatraemia is an adverse prognostic indicator (Rawson & Peto, 1990). The recent demonstration that vasopressin can stimulate $Ca^{2+}$-mobilisation in SCLC cell lines (Woll & Rozengurt, 1989*a*; Bunn *et al.*, 1990) implies that these cells have vasopressin receptors. This suggests that vasopressin could have an autocrine or paracrine function in these tumours.

### Tachykinins

The tachykinins, including substance P (11 amino acids) and substance K (ten amino acids), have similar activities but bind to distinct receptors. They are widely distributed in the brain, spinal cord and gut neurones. Their release causes local pain, smooth muscle contraction and vasodilatation, in addition to their systemic effects of stimulting natriuresis and salivation, and inhibiting pancreatic and biliary secretion. The substance K receptor was the first neuropeptide receptor to be cloned and sequenced (Masu *et al.*, 1987). The substance P receptor has considerable homology with it (Yokota *et al.*, 1989; Hershey & Krause, 1990). They are members of a group of receptors characterised by having seven helical transmembane domains, clustered to form a ligand-binding pocket, and coupled to $Ca^{2+}$-mobilising G-proteins (Dohlman *et al.*, 1987; Lefkowitz & Caron, 1988).

Substance P has been shown to have direct mitogenic effects on T-lymphocytes, mediated by specific receptors, at concentrations as low as 100 pM (Payan *et al.*, 1983). Tachykinins can also stimulate growth of human skin fibroblasts, arterial smooth muscle cells and keratinocytes (Nilsson *et al.*, 1985; Tanaka *et al.*, 1988). These, and earlier observations *in vivo*, have led to speculation that tachykinins mediate inflammation and wound healing (Payan, 1989). Substance P receptors are expressed in healing glia following neuronal injury (Mantyh *et al.*, 1989). Tachykinins have also been implicated in the pathogenesis of rheumatoid arthritis, as substance P stimulates prostaglandin E2 release and proliferation of synoviocytes (Lotz *et al.*, 1987).

Tachykinins are not known to be tumour growth factors, but they are secreted by some carcinoid tumours (Norheim *et al.*, 1986; Bishop *et al.*, 1989). The tachykinins have been demonstrated to mobilise $Ca^{2+}$ in SCLC cell lines, but not to stimulate growth (Takuwa *et al.*, 1990). An amphibian tachykinin, physalaemin (11 amino acids), is produced by some SCLC and appears to be able to inhibit SCLC growth, suggesting a possible regulatory role in this cancer (Lazarus *et al.*, 1983; Bepler *et al.*, 1987).

### Bradykinin

The nonapeptide bradykinin is generated in the plasma or tissues from high molecular weight precursors (kininogens) by the action of kallikreins, which are activated during proteolysis and clotting. It is rapidly degraded, so plasma concentrations are very low. Bradykinin is implicated in smooth muscle contraction, vasodilatation and vascular permeability. It is one of the most potent pain producing substances known, and its receptors are localised to the nocioceptive sensory pathways (Steranka *et al.*, 1988).

Bradykinin is a weak mitogen for human fibroblasts (Owen & Villereal, 1983; Coughlin *et al.*, 1985) but a potent mitogen for Swiss 3T3 cells (Woll & Rozengurt, 1988*a*). Acting synergistically with insulin in these cells, nanomolar concentrations of bradykinin achieve a response equivalent to that obtained with serum. Although bradykinin receptor subtypes can be distinguished pharmacologically, these do not correlate with functional differences, and the structures of the receptors are unknown. Bradykinin induces monovalent ion fluxes, transient protein kinase C activation, inositol phosphate production, $Ca^{2+}$-mobilisation, prostaglandin $E_2$ production and *myc* induction (Owen & Villereal, 1983; Coughlin *et al.*, 1985; Jackson *et al.*, 1987; Issandou & Rozengurt, 1990).

Bradykinin has not been demonstrated to act as a tumour growth factor. Its production however, has long been associated with the flushing caused by carcinoid tumours, which can secrete large quantities of vasoactive peptides (Oates *et al.*, 1964; Gustafsen *et al.*, 1987). Interestingly, [hydroxyprolyl$^3$]-bradykinin has been detected in the ascitic fluid of a patient with gastric cancer (Maeda *et al.*, 1988). Bradykinin has now been shown to stimulate $Ca^{2+}$ mobilisation in SCLC cell lines (Woll & Rozengurt, 1989*a*; 1990). Becuase it is produced at sites of tissue damage and rapidly inactivated, it could have local effects within tumours without being detected in serum by current assay methods.

### Opioids

Endogenous opioid peptides including the enkephalins, endorphins and dynorphins are widely distributed in the central nervous system. Multiple subtypes of receptors have been identified using a variety of agonists and antagonists. Because of their central role in pain transmission, opiate pharmacology has been studied in detail.

$\beta$-endorphin stimulates lymphocyte proliferation *in vitro*, although this effect may not be mediated directly by opiate receptors as it is not blocked by the opiate antagonist naltrexone (Gilman *et al.*, 1982). Dynorphins and enkephalins appear to be involved with vasopressin in the proliferative response of the rat marrow to haemorrhage (Feuerstein *et al.*, 1985). Further, $\beta$-endorphin has been implicated in the ability of newts to regenerate amputated limbs (Morley & Ensor, 1986).

SCLC cell lines contain opioid peptides and receptors (Roth & Barchas, 1985). Opioids have been reported both to inhibit and to stimulate SCLC growth (Davis *et al.*, 1989; Maneckjee & Minna, 1990). Opioids are also reported to inhibit the growth of breast cancer cells (Maneckjee *et al.*, 1990). Conversely, neuroblastoma xenograft growth can be inhibited by naltrexone (Zagon & McLaughlin, 1987), a reminder of the complex interactions between multifunctional growth factors (Sporn & Roberts, 1988)

### Vasoactive intestinal peptide (VIP)

This 28 amino acid peptide is found in large amounts in mammalian brain, and in gut mucosa and muscle, where it is localised to postganglionic nerves. It is also found in the salivary glands, pancreas, respiratory and urogenital tracts. Neural stimulation causes release of VIP, which binds to specific receptors. These receptors also bind related hormones (e.g. secretin, glucagon) with lower affinity. VIP induces relaxation of smooth muscle, vasodilatation and enhanced small intestinal and colonic secretion.

In Swiss 3T3 cells, VIP stimulates mitogenesis in the presence of insulin and cAMP phosphodiesterase inhibitors. In contrast to bombesin, vasopressin and bradykinin, VIP is a weak mitogen for these cells, and its effects are mediated by elevation of cAMP without $CA^{2+}$-mobilistion or protein kinase C activation (Zurier *et al.*, 1988). VIP also stimulates adenylate cyclase activity and cell proliferation in keratinocytes (Haegerstrand *et al.*, 1989).

VIP has been found in pancreatic, neural and cervical tumours and phaeochromocytomas (Bunnett *et al.*, 1984; Inoue *et al.*, 1984; Viale *et al.*, 1985), but is not known to be a growth factor for them. Pancreatic and intestinal VIP-secreting tumours (vipomas) present with the Verner-Morrison syndrome of watery diarrhoea, hypokalaemia and achlorhydria. Interestingly, this syndrome, and elevated serum VIP levels, were recently reported in a patient with SCLC (Noseda *et al.*, 1989). Some SCLC appear to have binding sites for VIP and VIP can stimulate growth of certain SCLC cell lines *in vitro*. This effect could be mediated by bombesin because exogeneous VIP stimulates bombesin/GRP secretion (Bepler *et al.*, 1988).

### Further neuropeptide growth factors

The number of neuropeptides shown to be mitogenic is rapidly increasing. Serotonin (5-hydroxy tryptamine) has long been known to be a component of the carcinoid flush and is also secreted by some SCLC (Horai *et al.*, 1973; Maton, 1988). Its receptor has been cloned and belongs to the class of G-protein linked receptors with seven helical transmembrane domains (Julius *et al.*, 1988). Expression of the serotonin receptor in fibroblasts leads to malignant transformation, suggesting that it can act as a proto-oncogene (Julius *et al.*, 1989). Whether this is significant in any human cancer is unknown. The angiotensin receptor, which has a similar structure, is also encoded by an oncogene, *mas* (Young *et al.*, 1986; Jackson *et al.*, 1988).

The recently described neuropeptide endothelin, and related vasoconstrictor peptides, stimulate DNA synthesis by a $Ca^{2+}$ dependent pathway in a variety of cell types including fibroblasts, smooth muscle and glial cells (Komuro *et al.*, 1988; Yanigasawa *et al.*, 1988; Takuwa *et al.*, 1989; Fabregat & Rozengurt, 1990). Endothelin is produced by breast, pancreas and colon cancer cell lines, but it is not yet known whether these, or surrounding stromal cells, are stimulated by it (Kusuhara *et al.*, 1990).

**Table I** Peptides and hormones secreted by SCLC

| | |
|---|---|
| Adrenocorticotrophin | Lipotropin |
| Atrial natriuretic peptide | Neurotensin |
| Bombesin/GRP | Opioid peptides |
| Calcitonin | Oxytocin |
| Calcitonin gene related peptide | Parathyroid hormone |
| Cholecystokinin | Physalaemin |
| Chorionic gonadotrophin | Prolactin |
| Estradiol | Serotonin |
| Follicle stimulating hormone | Somatostatin |
| Gastrin | Substance K |
| Glucagon | Substance P |
| Granulocyte colony stimulating factor | Transferrin |
| Growth hormone | Vasopressin |
| Growth hormone releasing factor | VIP |
| Insulin-like growth factor-I | |

### Therapeutic implications

The recognition that many neuropeptides can act as growth factors, the identification of these peptides and their receptors in human cancers, and the discovery that some are encoded by oncogenes (Young *et al.*, 1986; Jackson *et al.*, 1988; Julius *et al.*, 1989), have led to speculation that neuropeptides are important regulators of tumur growth. It has long been known that tumours can secrete diverse peptides, in a heterogeneous fashion. Only recently however have multiple receptors been demonstrated on cancer cells, prompting the suggestion that growth of these tumours is regulated by multiple factors acting in a paracrine or autocrine manner (Woll & Rozengurt, 1989*a*).

SCLC is the best example of this model. It can secrete a wide variety of ectopic peptides and hormones (Table I). Many of these have been shown to act as growth factors for other cell types, *in vitro* and *in vivo*. The demonstration of receptors for bombesin/GRP, bradykinin, cholecystokinin, galanin, gastrin, neurotensin, tachykinins and vasopressin on SCLC permits speculation that these diverse peptides may contribute to multiple growth loops. Direct evidence of growth stimulation in SCLC is at present available only for bombesin/GRP, neurotensin and β-endorphin, but it is likely that further neuropeptide mitogens will soon be added to this list. Other tumuors for which neuropeptide growth factors may be important include the neuroendocrine cancers (e.g. carcinoids, medullary carcinoma of thyroid) and adenocarcinomas of stomach, colon, breast and prostate.

The model proposed, of tumour growth regulation by multiple neuropeptide growth factors, has implications for future therapeutic strategies. Attempts to produce highly specific antibodies and antagonists to these growth factors are unlikely to be successful except in a minority of cases. At best, 30–60% of cells may express receptors for any individual peptide (Bunn *et al.*, 1990) but all or most tumours will express receptors for multiple peptides (Woll & Rozengurt, 1989*a*). Possible strategies to exploit this knowledge include targetting a signalling process common to the diverse peptides, attacking an enzyme essential to growth factor production, and developing broad-spectrum receptor antagonists. Elucidation of mitogenic signalling pathways has identified a number of intracellular messengers used by many mitogens, such as protein kinase C activation or ion fluxes, that could be targets for novel therapies (Woll & Rozengurt, 1989*b*). Broad-spectrum neuropeptide antagonists, that appear to act on a class of G-protein-linked, $Ca^{2+}$-mobilising receptors, have already been shown to block the early events stimulated by diverse neuropeptides and to inhibit SCLC growth *in vitro* (Woll & Rozengurt, 1988*b*, 1990). Increasing knowledge of the actions of neuropeptide growth factors in tumours will undoubtedly lead to innovations in cancer treatment.

### References

ALEXANDER, R.W., UPP, J.R., POSTON, G.J., GUPTA, V., TOWNSEND, C.M. & THOMPSON, J.C. (1988). Effects of bombesin on growth of human small cell lung carcinoma *in vivo*. *Cancer Res.*, **48**, 1439.

BATTEY, J.F., WAY, J.M., CORJAY, M.H. & 7 others (1991). Molecular cloning of the bombesin/GRP receptor from Swiss 3T3 cells. *Proc. Natl Acad. Sci. USA*, (in press).

BEPLER, G., CARNEY, D.N., GAZDAR, A.F. & MINNA, J.D. (1987). *In vitro* growth inhibition of human small cell lung cancer by physalaemin. *Cancer Res.*, **47**, 2371.

BEPLER, G., ROTSCH, M., JAQUES, G. & 5 others (1988). Peptides and growth factors in small cell lung cancer: production, binding sites and growth effects. *J. Cancer Res. Clin. Oncol.*, **114**, 235.

BETTON, G.R., DORMER, C.S., WELLS, T., PERT, P., PRICE, C.A. & BUCKLEY, P. (1988). Gastric ECL-cell hyperplasia and carcinoids in rodents following chronic administration of $H_2$-antagonists SK&F 93479 and oxymetidine and omeprazole. *Toxicol. Pathol.*, **16**, 288.

BISHOP, A.E., HAMID, Q.A., ADAMS, C. & 13 others (1989). Expression of tachykinins by ileal and lung carcinonoid tumors assessed by combined *in situ* hybridization, immunocytochemistry, and radioimmunoassay. *Cancer*, **63**, 1129.

BOER, G.J. (1985). Vasopressin and brain development: studies using the Brattleboro rat. *Peptides*, **6** (Suppl. 1), 49.

BOLOGNA, M., FESTUCCIA, C., MUZI, P., BIORDI, L. & CIOMEI, M. (1989). Bombesin stimulates growth of human prostatic cancer cells *in vitro*. *Cancer*, **63**, 1714.

BOSTWICK, D.G. & BENSCH, K.G. (1985). Gastrin releasing peptide in human neuroendocrine tumours. *J. Pathol.*, **147**, 237.

BUNN, P.A., DIENHART, D.G., CHAN, D. & 4 others (1990). Neuropeptide stimulation of calcium flux in human lung cancer cells: delineation of alternative pathways. *Proc. Natl Acad. Sci USA*, **87**, 2162.

BUNNETT, N.W., REEVE, J.R., DIMALINE, R., SHIVELY, J.E., HAWKE, D. & WALSH, J.H. (1984). The isolation and sequence analysis of vasoactive intestinal peptide from a ganglioneuroblastoma. *J. Clin. Endocrinol. Metab.*, **59**, 1133.

CARNEY, D.N., CUTTITTA, F., MOODY, T.W. & MINNA, J.D. (1987). Selective stimulation of small cell lung cancer clonal growth by bombesin and gastrin-releasing peptide. *Cancer Res.*, **47**, 821.

CHAN, D., STEWART, J., VAVREK, R. & 6 others (1990). Effects of bradykinin and bradykinin antagonists on intracellular calcium concentrations in human lung cancer cells. *Proc. Am. Ass. Cancer Res.*, **31**, 54.

COFFER, A., FABREGAT, I., SINNETT-SMITH, J. & ROZENGURT, E. (1990). Solubilization of the bombesin receptor from Swiss 3T3 cell membranes. Functional association to a guanine nucleotide regulatory protein. *FEBS Lett.*, **263**, 80.

COLLINS, M.K.L. & ROZENGURT, E. (1983). Vasopressin induces selective desensitization of its mitogenic response in Swiss 3T3 cells. *Proc. Natl Acad. Sci. USA*, **80**, 1924.

COUGHLIN, S.R., LEE, W.M.F., WILLIAMS, P.W., GIELS, G.M. & WILLIAMS, L.T. (1985). c-*myc* gene expression is stimulated by agents that activate protein kinase C and does not account for the mitogenic effect of PDGF. *Cell*, **43**, 243.

CUTTITTA, F., CARNEY, D.N., MULSHINE, J. & 4 others (1985). Bombesin-like peptides can function as autocrine growth factors in human small-cell lung cancer. *Nature*, **316**, 826.

DAVIS, T.P., BURGESS, H.S., CROWELL, S., MOODY, T.W., CULLING-BERLUND, A. & LIU, R.H. (1989). β-endophin and neurotensin stimulate *in vitro* clonal growth of human SCLC cells. *Eur. J. Pharmacol.*, **161**, 283.

DEMBINSKI, A.B. & JOHNSON, L.R. (1979). Growth of pancreas and gastrointestinal mucosa in antrectomized and gastrin-treated rats. *Endocrinology*, **105**, 769.

DOHLMAN, H.G., CARON, M.G. & LEFKOWITZ, R.J. (1987). A family of receptors coupled to guanine nucleotide regulatory proteins. *Biochemistry*, **26**, 2657.

DOUGLAS, B.R., WOUTERSEN, R.A., JANSEN, J.B.M.J., ROVATI, L.C. & LAMERS, C.B.H.W. (1989). Study into the role of cholecystokinin in bombesin-stimulated pancreatic growth in rats and hamsters. *Eur. J. Pharmacol.*, **161**, 209.

EKMAN, L., HANSSON, E., HAVU, N., CARLSSON, E. & LUNDBERG, C. (1985). Toxicological studies on omeprazole. *Scand. J. Gastroenterol.*, **20** (Suppl. 108), 53.

ERISMAN, M.D., LINNOILA, R.I., HERNANDEZ, O., DIAUGUSTINE, R.P. & LAZARUS, L.H. (1982). Human lung small-cell carcinoma contains bombesin. *Proc. Natl Acad. Sci. USA*, **79**, 2379.

FABREGAT, I. & ROZENGURT, E. (1990). Vasoactive intestinal constrictor a novel peptide, shares a common receptor with endothelin-1 and stimulates $Ca^{2+}$ mobilization and DNA synthesis in Swiss 3T3 cells. *Biochem. Biophys. Res. Commun.*, **167**, 161.

FEUERSTEIN, G., MOLINEAUX, C.J., ROSENBERGER, J.G., ZERBE, R.L., COX, B.M. & FADEN, A.I. (1985). Hemorrhagic shock and the central vasopressin and epioid peptide system of rats. *Am. J. Physiol.*, **249**, E244.

GAZDAR, A.F. & CARNEY, D.N. (1984). Endocrine properties of small cell carcinoma of the lung. In *The Endocrine Lung in Health and Disease*, Becker, K.L. & Gazdar, A.F. (eds) p. 501. W.B. Saunders: London.

GIACCHETTI, S., GAUVILLE, C., DE CREMOUX, P. & 5 others (1990). Characterization, in some human breast cell lines, of gastrin-releasing peptide-like receptors which are absent in normal breast epithelial cells. *Int. J. Cancer*, **46**, 293.

GILMAN, S.C., SCHWATRZ, J.M., MILNER, R.J., BLOOM, F.E. & FELDMAN, J.D. (1982). β-Endorphin enhances lymphocyte proliferative responses. *Proc. Natl Acad. Sci. USA*, **79**, 4226.

GOEDERT, M., REEVE, J.G., EMSON, P.C. & BLEEHEN, N.M. (1984). Neurotensin in human small cell lung carcinoma. *Br. J. Cancer*, **50**, 179.

GUSTAFSEN, J., BOESBY, S., NIELSEN, F. & GIESE, J. (1987). Bradykinin in the carcinoid syndrome. *Gut*, **28**, 1417.

HAARSTAD, H., STEFFENSRUD, S., WINNBERG, A. & PETERSEN, H. (1986). The trophic effect on the pancreas of long-term continuous intravenous infusion of secretin and a cholecystokinin-like peptide in rats. *Scand. J. Gastroenterol.*, **21**, 589.

HÆGERSTRAND, A., JONZON, B., DALSGAARD, C.-J. & NILSSON, J. (1989). Vasoactive intetinal polypeptide stimulates cell proliferation and adenylate cyclase activity of cultured human keratinocytes. *Proc. Natl Acad. Sci. USA*, **86**, 5993.

HANLEY, M.R. (1985). Neuropeptides as mitogens. *Nature*, **315**, 14.

HANSEN, O.H., PEDERSEN, T., LARSEN, J.K. & REHFELD, J.F. (1976). Effect of gastric mucosal cell proliferation in man. *Gut*, **17**, 536.

HEIKKILA, R., TREPEL, J.B., CUTTITTA, F., NECKERS, L.M. & SAUSVILLE, E.A. (1987). Bombesin-related peptides induce calcium mobilization in a subset of human small cell lung cancer cell lines. *J. Biol. Chem.*, **262**, 16356.

HERSHEY, A.D. & KRAUSE, J.E. (1990). Molecular characterization of a functional cDNA encoding the rat substance P receptor. *Science*, **247**, 958.

HOOSEIN, N.M., KIENER, P.A., CURRY, R.C., ROVATI, L.C., MCGILBRA, D.K. & BRATTAIN, M.G. (1988). Antiproliferative effects of gastrin receptor antagonists and antibodies to gastrin on human colon carcinoma cell lines. *Cancer Res.*, **48**, 7179.

HOOSEIN, N.M., KIENER, P.A., CURRY, R.C. & BRATTAIN, M.G. (1990). Evidence for autocrine growth stimulation of cultured colon tumor cells by a gastrin/cholecystokinin-like peptide. *Exp. Cell Res.*, **186**, 15.

HORAI, T., NISHIHARA, H., TATEISHI, R., MATSUDA, M. & HATTORI, S. (1973). Oat cell carcinoma of the lung simultaneously producing ACTH and serotonin. *J. Clin. Endocrinol. Metab.*, **37**, 212.

HUNT, N.H., PERRIS, A.C. & SANDFORD, P.A. (1977). Role of vasopressin in the mitotic response of rat bone marrow cells to haemorrhage. *J. Endocrinol.*, **72**, 5.

INOUE, T., YAMAGUCHI, K., SUZUKI, H., ABE, K. & CHIHARA, T. (1984). Production of immunoreactive-polypeptide hormones in cervical carcinoma. *Cancer*, **53**, 1509.

ISACKE, C.M., MEISENHELDER, J., BROWN, K.D., GOULD, K.L., GOULD, S.J. & HUNTER, T. (1986). Early phosphorylation events following the treatment of Swiss 3T3 cells with bombesin and the mammalian bombesin-related peptide, gastrin-releasing peptide. *EMBO J*, **5**, 2889.

ISSANDOU, M. & ROZENGURT, E. (1990). Bradykinin transiently activates protein kinase C in Swiss 3T3 cells. Distinction from activation by bombesin and vasopressin. *J. Biol. Chem.*, **265**, 11890.

JACKSON, T.R., HALLAM, T.J., DOWNES, C.P. & HANLEY, M.R. (1987). Receptor coupled events in bradykinin action: rapid production of inositol phosphates and regulation of cytosolic free $Ca^{2+}$ in a neural cell line. *EMBO J.*, **6**, 49.

JACKSON, T.R., BLAIR, L.A.C., MARSHALL, J., GOEDERT, M. & HANLEY, M. (1988). The *mas* oncogene encodes an angiotensin receptor. *Nature*, **335**, 437.

JULIUS, D., LIVELLI, T.J., JESSELL, T.M. & AXEL, R. (1989). Ectopic expression of the serotonin 1c receptor and the triggering of malignant transformation. *Science*, **244**, 1057.

JULIUS, D., MACDERMOTT, A.B., AXEL, R. & JESSELL, T.M. (1988). Molecular characterization of a functional cDNA encoding the serotonin 1c receptor. *Science*, **241**, 558.

KADO-FONG, H. & MALFROY, B. (1989). Effects of bombesin on human small cell lung cancer cells: evidence for a subset of bombesin non-responsive cell lines. *J. Cell Biochem.*, **40**, 431.

KOBORI, O., VUILLOT, M.-T. & MARTIN, F. (1982). Growth responses of rat stomach cancer cells to gastro-entero-pancreatic hormones. *Int. J. Cancer*, **30**, 65.

KOMURO, I., KURIHARA, H., SUGIYAMA, T., TAKAHU, F. & YAZAKI, Y. (1988). Endothelin stimulates c-*fos* and c-*myc* expression and proliferation of vascular smooth muscle cells. *FEBS Lett.*, **238**, 249.

KRIS, R.M., HAZAN, R., VILLINES, J., MOODY, T.W. & SCHLESSINGER, J. (1987). Identification of the bombesin receptor on murine and human cells by cross-linking experiments. *J. Biol. Chem.*, **262**, 11215.

KUSUHARA, M., YAMAGUCHI, K., NAGASAKI, K. & 6 others (1990). Production of endothelin in human cancer cell lines. *Cancer Res.*, **50**, 3257.

LAMERS, C.B.H.W. & JANSEN, J.B.M.J. (1988). Role of gastrin and cholecystokinin in tumours of the gastrointestinal tract. *Eur. J. Cancer Clin. Oncol.*, **24**, 267.

LAYTON, J.E., SCANLON, D.B., SOVENY, C. & MORSTYN, G. (1988). Effects of bombesin antagonists on the growth of small cell lung cancer cells *in vitro*. *Cancer Res.*, **48**, 4783.

LAZARUS, L.H., DIAUGUSTINE, R.P., JAHNKE, G.D. & HERNANDEZ, O. (1983). Physalaemin: an amphibian tachykinin in human lung small-cell carcinoma. *Science*, **219**, 79.

LEFKOWITZ, R.J. & CARON, M.G. (1988). Adrenergic receptors: models for the study of receptors coupled to guanine nucleotide regulatory proteins. *J. Biol. Chem.*, **263**, 4993.

LEHY, T., ACCARY, J.P., LABEILLE, D. & DUBRASQUET, M. (1983). Chronic administration of bombesin stimulates antral gastrin cell proliferation in the rat. *Gastroenterology*, **84**, 914.

LETTERIO, J.J., COUGHLIN, S.R. & WILLIAMS, L.T. (1986). Pertussis-toxin-sensitive pathway in the stimulation of c-*myc* expression and DNA synthesis by bombesin. *Science*, **234**, 1117.

LEZOCHE, E., BASSO, N. & SPERANZA, V. (1981). Actions of bombesin in man. In *Gut Hormones*, Bloom, S.R. & Polak, J.M. (eds) p. 419. Churchill Livingstone: London.

LHOSTE, E.F., APRAHAMIAN, M., BALBONI, G. & DAMGÉ, C. (1989). Evidence for a direct trophic effect of bombesin on the mouse pancreas: *in vivo* and cell culture studies. *Regul. Peptides*, **24**, 45.

LHOSTE, E.F. & LONGNECKER, D.S. (1987). Effect of bombesin and caerulein on early stages of carcinogenesis induced by azaserine in the rat pancreas. *Cancer Res.*, **47**, 3273.

LOPEZ-RIVAS, A., MENDOZA, S.A., NÅNBERG, E., SINNETT-SMITH, J. & ROZENGURT, E. (1987). The $Ca^{2+}$-mobilizing actions of platelet-derived growth factor differ from those of bombesin and vasopressin in Swiss 3T3 cells. *Proc. Natl Acad. Sci. USA*, **84**, 5768.

LOTZ, M., CARSON, D.A. & VAUGHAN, J.H. (1987). Substance P activation of rheumatoid synoviocytes: neural pathway in pathogenesis of arthritis. *Science*, **235**, 893.

MADSEN, O.D., LARSSON, L.-I., REHFELD, J.F. & 4 others (1986). Cloned cell lines from a transplantable islet cell tumor are heterogeneous and express cholecystokinin in addition to islet hormones. *J. Cell Biol.*, **103**, 2025.

MAEDA, H., MATSUMARA, Y. & KATO, H. (1988). Purification and identification of [hydroxyprolyl$^3$]bradykinin in ascitic fluid from a patient with cancer. *J. Biol. Chem.*, **263**, 16051.

MANECKJEE, R., BISWAS, R. & VONDEHAAR, B.K. (1990). Binding of opioids to human MCF-7 breast cancer cells and their effects on growth. *Cancer Res.*, **50**, 2234.

MANECKJEE, R. & MINNA, J.D. (1990). Opioid and nicotine receptors effect growth regulation of human lung cancer cell lines. *Proc. Natl Acad. Sci. USA*, **87**, 3294.

MANTYH, P.W., JOHNSON, D.J., BOEHMER, C.G. & 5 others (1989). Substance P receptor binding sites are expressed by glia *in vivo* after neuronal injury. *Proc. Natl Acad. Sci. USA*, **86**, 5193.

MASU, Y., NAKAYAMA, K., TAMAKI, H., HARADA, Y., KUNO, M. & NAKANISHI, S. (1987). cDNA cloning of bovine substance K receptor through oocyte expression system. *Nature*, **329**, 836.

MATON, P.N. (1988). The carcinoid syndrome. *J. Am. Med. Assoc.*, **260**, 1602.

MAURER, L.H., O'DONNELL, J.F., KENNEDY, S., FAULKNER, C.S., RIST, K. & NORTH, W.G. (1983). Human neurophysins in carcinoma of the lung: relation to histology, disease stage, response, rate, survival, and syndrome of inappropriate antidiuretic hormone secretion. *Cancer Treat. Rep.*, **67**, 971.

MENDOZA, S.A., SCHNEIDER, J.A., LOPEZ-RIVAS, A., SINNETT-SMITH, J.W. & ROZENGURT, E. (1986). Early events elicited by bombesin and structurally related peptides in quiescent Swiss 3T3 cells. II. Changes in $Na^+$ and $Ca^{2+}$ fluxes, $Na^+/K^+$ pump activity, and intracellular pH. *J. Cell. Biol.*, **102**, 2223.

MILLAR, J.B.A. & ROZENGURT, E. (1988). Bombesin enhancement of cAMP accumulation in Swiss 3T3 cells: evidence of a dual mechanism of action. *J. Cell. Physiol.*, **137**, 214.

MILLAR, J.B.A. & ROZENGURT, E. (1990). Arachidonic acid release by bombesin: a novel post-receptor target for heterologous mitogenic desensitization. *J. Biol. Chem.*, **265**, 19973.

MOODY, T.W., CARNEY, D.N., CUTTITTA, F., QUATTROCHI, K. & MINNA, J.D. (1985*a*). High affinity receptors for bombesin/GRP-like peptides on human small cell lung cancer. *Life Sci.*, **37**, 105.

MOODY, T.W., CARNEY, D.N., KORMAN, L.Y., GAZDAR, A.F. & MINNA, J.D. (1985*b*). Neurotensin is produced by and secreted from classic small cell lung cancer cells. *Life Sci.*, **36**, 1727.

MOODY, T.W., MURPHY, A., MAHMOUD, S. & FISKUM, G. (1987). Bombesin-like peptides elevate cytosolic calcium in small cell lung cancer cells. *Biochem. Biophys. Res. Commun.*, **147**, 189.

MOODY, T.W., PERT, C.B., GAZDAR, A.F., CARNEY, D.N. & MINNA, J.D. (1981). High levels of intracellular bombesin characterize small-cell lung carcinoma. *Science*, **214**, 1246.

MORLEY, J.S. & ENSOR, D.M. (1986). Neurotrophic effects of beta-endorphin C-terminal tetrapeptide (MPF). *Neuropeptides*, **8**, 45.

NILSSON, J., VON EULER, A.M. & DALSGAARD, C.J. (1985). Stimulation of connective tissue cell growth by substance P and substance K. *Nature*, **315**, 61.

NORHEIM, I., THEODORSSON-NORHEIM, E., BRODIN, E. & OBERG, K. (1986). Tachykinins in carcinoid tumours: their use as a tumour marker and possible role in the carcinoid flush. *J. Clin. Endocrinol. Metab.*, **63**, 605.

NORTH, W.G., MAURER, L.H., VALTIN, H. & O'DONNELL, J.F. (1980). Human neurophysins as potential tumor markers for small cell carcinoma of the lung: application of specific radioimmunoassay. *J. Clin. Endocrinol. Metab.*, **51**, 892.

NOSEDA, A., FUSS, M., DE NUTTE, N., COGAN, E., SCHMERBER, J. & CORVILAN, J. (1989). Vipoma syndrome simultaneously occurring with small-cell carcinoma of the lung. *Arch. Intern. Med.*, **149**, 1223.

OATES, J.A., MELMON, K., SJOERDSMA, A., GILLESPIE, L. & MASON, D.T. (1964). Release of a kinin peptide in the carcinoid syndrome. *Lancet*, **i**, 514.

OWEN, N.E. & VILLEREAL, M.L. (1983). Lys-bradykinin stimulates $Na^+$ influx and DNA synthesis in cultured human fibroblasts. *Cell*, **32**, 979.

PATEL, K.V. & SCHREY, M.P. (1990). Activation of inositol phospholipid signalling and $Ca^{2+}$ efflux in human breast cancer cells by bombesin. *Cancer Res.*, **50**, 235.

PAYAN, D.G. (1989). Neuropeptides and inflammation: the role of substance P. *Annu. Rev. Med.*, **40**, 341.

PAYAN, D.G., BREWSTER, D.R. & GOETZL, E.J. (1983). Specific stimulation of human T lymphocytes by substance P. *J. Immunol.*, **131**, 1613.

POYNTER, D., PICK, C.R., HARCOURT, R.A. & 6 others (1985). Association of long lasting unsurmountable histamine $H_2$ blockade and gastric carcinoid tumours in the rat. *Gut*, **26**, 1284.

RASMUSSEN, T.N., JØRGENSEN, P.E., ALMDAL, T., POULSEN, S.S. & OLSEN, P.S. (1990). Effect of gastrin on liver regeneration after partial hepatectomy in rats. *Gut*, **31**, 92.

RAWSON, N.S.B. & PETO, J. (1990). An overview of prognostic factors in small cell lung cancer. *Br. J. Cancer*, **61**, 597.

REHFELD, J.F., LINDHOLM, J., ANDERSEN, B.N. & 4 others (1987). Pituitary tumors containing cholecystokinin. *N. Engl. J. Med.*, **316**, 1244.

REHFELD, J.F., BARDRAM, L. & HILSTED, L. (1989). Gastrin in human bronchogenic carcinomas: constant expression but variable processing of progastrin. *Cancer Res.*, **49**, 2840.

RODRIGUEZ-PENA, A. & ROZENGURT, E. (1986). Vasopressin rapidly stimulates protein kinase C in quiescent Swiss 3T3 cells. *J. Cell Physiol.*, **129**, 124.

ROTH, K.A. & BARCHAS, J.D. (1986). Small cell carcinoma cell lines contain opioid peptides and receptors. *Cancer*, **57**, 769.

ROZENGURT, E. (1986). Early signals in the mitogenic response. *Science*, **234**, 161.

ROZENGURT, E., LEGG, A. & PETTICAN, P. (1979). Vasopressin stimulation of mouse 3T3 cell growth. *Proc. Natl Acad. Sci. USA*, **76**, 1284.

ROZENGURT, E., RODRIGUEZ-PENA, A. & SMITH, K.A. (1983). Phorbol esters, phospholipase C, and growth factors rapidly stimulate the phosphorylation of a $M_r$ 80,000 protein in intact quiescent 3T3 cells. *Proc. Natl Acad. Sci. USA*, **80**, 7244.

ROZENGURT, E. & SINNETT-SMITH, J. (1983). Bombesin stimulation of DNA synthesis and cell division in cultures of Swiss 3T3 cells. *Proc. Natl Acad. Sci. USA*, **80**, 2936.

ROZENGURT, E. & SINNETT-SMITH, J. (1988). Early signals underlying the induction of the c-*fos* and c-*myc* genes in quiescent fibroblasts: studies with bombesin and other growth factors. *Progr. Nucleic Acid Res. Mol. Biol.*, **35**, 261.

RUSSELL, W.E. & BUCHER, N.L.R. (1983). Vasopressin modulates liver regeneration in the Brattleboro rat. *Am. J. Physiol.*, **245**, G321.

RYBERG, B., AXELSON, J., HÅKANSON, R., SUNDLER, F. & MATTSON, H. (1990). Trophic effects of continuous infusion of [Lys$^{15}$]-gastrin-17 in the rat. *Gastroenterology*, **98**, 33.

SAUSVILLE, E., CARNEY, D. & BATTEY, J. (1985). The human vasopressin gene is linked to the oxytocin gene and is selectively expressed in a cultured lung cancer cell line. *J. Biol. Chem.*, **260**, 10236.

SCHWARTZ, W.B., BENNETT, W., CURELOP, S. & BARTTER, F.C. (1957). A syndrome of renal sodium loss and hyponatraemia probably resulting from inappropriate secretion of antidiuretic hormone. *Am. J. Med.*, **23**, 529.

SEGLEN, P.O., SKOMEDAL, H., SAETER, G., SCHWARZE, P.E. & NESLAND, J.M. (1989). Neuroendocrine dysdifferentiation and bombesin production in carcinogen-induced hepatocellular rat tumours. *Carcinogenesis*, **10**, 21.

SINGH, P., WALKER, J.P., TOWNSEND, C.M. & THOMPSON, J.C. (1986). Role of gastrin and gastrin receptors on the growth of a transplantable mouse colon carcinoma (MC-26) in Balb/c mice. *Cancer Res.*, **46**, 1612.

SINNETT-SMITH, J., LEHMANN, W. & ROZENGURT, E. (1990). Bombesin receptor in membranes from Swiss 3T3 cells. Binding characteristics, affinity labelling and modulation by guanine nucleotides. *Biochem. J.*, **265**, 485.

SMITH, J.P., WOOD, J.G. & SOLOMON, T.E. (1989). Elevated gastrin levels in patients with colon cancer or adenomatous polyps. *Dig. Dis. Sci.*, **34**, 171.

SOLOMON, T.E., MORRISET, J., WOOD, J.G. & BUSSJAEGER, L.J. (1987). Additive interaction of pentagastrin and secretin on pancreatic growth in rats. *Gastroenterology*, **92**, 429.

SORENSON, G.D., PETTENGILL, O.S., BRINCK-JOHNSEN, T., CATE, C.C. & MAURER, L.H. (1981). Hormone production by cultures of small-cell carcinoma of the lung. *Cancer*, **47**, 1289.

SPINDEL, E.R., CHIN, W.W., PRICE, J., REES, L.H., BESSER, G.M. & HABENER, J.F. (1984). Cloning and characterization of cDNAs encoding human gastrin-releasing peptide. *Proc. Natl Acad. Sci. USA*, **81**, 5699.

SPINDEL, E.R., SUNDAY, M.E., HOFLER, H., WOLFE, H.J., HABENER, J.F. & CHIN, W.W. (1987). Transient elevation of messenger RNA encoding gastrin-releasing peptide, a putative pulmonary growth factor in human fetal lung. *J. Clin. Invest.*, **80**, 1172.

SPORN, M.B. & ROBERTS, A.B. (1988). Peptide growth factors are multifunctional. *Nature*, **332**, 217.

STALEY, J., FISKUM, G. & MOODY, T.W. (1989*a*). Cholecystokinin elevates cytosolic calcium in small cell lung cancer cells. *Biochem. Biophys. Res. Commun.*, **163**, 605.

STALEY, J., FISKUM, G., DAVIS, T.P. & MOODY,. T.W. (1989*b*). Neurotensin elevates cytosolic calcium in small cell lung cancer cells. *Peptides*, **10**, 1217.

STERANKA, L.R., MANNING, D.C., DEHAAS, C.J. & 6 others (1988). Bradykinin as a pain mediator: receptors are localised to sensory neurons, and antagonists have analgesic actions. *Proc. Natl Acad. Sci. USA*, **85**, 3245.

SUMIYOSHI, H., YASUI, W., OCHIAI, A. & TAHARA, E. (1984). Effects of gastrin on tumour growth and cyclic nucleotide metabolism in xenotransplantable human gastric and colonic carcinomas in nude mice. *Cancer Res.*, **44**, 4276.

SUZUKI, M., YAMAGUCHI, K., ABE, K. & 9 others (1987). Detection of gastrin-releasing peptide mRNA in small cell lung carcinomas and medullary thyroid carcinomas using synthetic oligodeoxyribonucleotide probes. *Jpn. J. Clin. Oncol.*, **17**, 157.

TAKUWA, N., TAKUWA, Y., BOLLAG, W.E. & RASMUSSEN, H. (1987). The effects of bombesin on polyphosphoinositide and calcium metabolism in Swiss 3T3 cells. *J. Biol. Chem.*, **262**, 182.

TAKUWA, N., TAKUWA. Y., OHUE, Y. & 5 others (1990). Stimulation of calcium mobilization but not proliferation by bombesin and tachykinin neuropeptides in human small cell lung cancer cells. *Cancer Res.*, **50**, 240.

TAKUWA, N., TAKUWA, Y., YANIGASAWA, M., YAMASHITA, K. & MASAKI, T. (1989). A novel vasoactive peptide endothelin stimulates mitogenesis through inositol lipid turnover in Swiss 3T3 fibroblasts. *J. Biol. Chem.*, **264**, 7856.

TANAKA, T., DANNO, K., IKAI, K., IMAMURA, S. (1988). Effects of substance P and substance K on the growth of cultured keratinocytes. *J. Invest. Dermatol.*, **90**, 399.

TREPEL, J.B., MOYER, J.D., HEIKKILA, R. & SAUSVILLE, E.A. (1988). Modulation of bombesin-induced phosphatidylinositol hydrolysis in a small-cell lung-cancer cell line. *Biochem. J.*, **255**, 403.

UPP, J.R., SINGH, P., TOWNSEND, C.M. & THOMPSON, J.C. (1989). Clinical significance of gastrin receptors in human colon cancers. *Cancer Res.*, **49**, 488.

VIALE, G., DELL'ORTO, P., MORO, E., COZZAGLIO, L. & COGGI, G. (1985). Vasoactive intestinal polypeptide-, somatostatin-, and calcitonin-producing adrenal pheochromocytoma associated with the watery diarrhea (WDHA) syndrome. *Cancer*, **55**, 1099.

WATSON, S., DURRANT, L. & MORRIS, D. (1989). Gastrin: growth enhancing effects on human gastric and colonic tumour cells. *Br. J. Cancer*, **59**, 554.

WEBER, C.J., O'DORISIO, T.M., McDONALD, T.J., HOWE, B., KOSCHITZKY, T. & MERRIAM, L. (1989). Gastrin-releasing peptide-, calcitonin gene-related peptide-, and calcitonin-like immunoreactivity in human breast cyst fluid and gastrin-releasing peptide-like immunoreactivity in human breast carcinoma cell lines. *Surgery*, **106**, 1134.

WEBER, S., ZUCKERMAN, J.E., BOSTWICK, D.G., BENSCH, K.G., SIKIC, B.I. & RAFFIN, T.A. (1985). Gastrin-releasing peptide is a selective mitogen for small cell lung carcinoma *in vitro*. *J. Clin. Invest.*, **75**, 306.

WHARTON, J., POLAK, J.M., BLOOM, S.R. & 4 others (1978). Bombesin-like immunoreactivity in the lung. *Nature*, **273**, 769.

WILLEY, J.C., LECHNER, J.F. & HARRIS, C.C. (1984). Bombesin and the C-terminal tetradecapeptide of gastrin-releasing peptide are growth factors for normal human bronchial epithelial cells. *Exp. Cell Res.*, **153**, 245.

WOLL, P.J. & ROZENGURT, E. (1988*a*). Two classes of antagonist interact with receptors for the mitogenic neuropeptides bombesin, bradykinin and vasopressin. *Growth Factors*, **1**, 75.

WOLL, P.J. & ROZENGURT, E. (1988*b*). [DArg$^{1}$, DPhe$^{5}$, DTrp$^{7,9}$,Leu$^{11}$]- substance P, a potent bombesin antagonist in murine Swiss 3T3 cells, inhibits the growth of human small cell lung cancer cells *in vitro*. *Proc. Natl Acad. Sci. USA*, **85**, 1859.

WOLL, P.J. & ROZENGURT, E. (1989*a*). Multiple neuropeptides mobilise calcium in small cell lung cancer: effects of vasopressin, bradykinin, cholecystokinin, galanin and neurotensin. *Biochem. Biophys. Res. Commun.*, **164**, 66.

WOLL, P.J. & ROZENGURT, E. (1989*b*). Therapeutic implications of growth factors in small cell lung cancer. *Lung Cancer*, **5**, 287.

WOLL, P.J. & ROZENGURT, E. (1990). A neuropeptide antagonist that inhibits the growth of small cell lung cancer *in vitro*. *Cancer Res.*, **50**, 3968.

WOOD, S.M., WOOD, J.R., GHATEL, M.A., LEE, Y.C., O'SHAUGHNESSY, D. & BLOOM, S.R. (1981). Bombesin, somatostatin and neurotensin-like immunoreactivity in bronchial carcinoma. *J. Clin. Endocrinol. Metab.*, **53**, 1310.

YANIGASAWA, M., KURIHARA, H., KIMURA, S. & 6 others (1988). A novel potent vasoconstrictor peptide produced by vascular endothelial cells. *Nature*, **332**, 411.

YODER, D.G. & MOODY, T.W. (1987). High affinity binding of cholecystokinin to small cell lung cancer cells. *Peptides*, **8**, 103.

YOKOTA, Y., SASAI, Y., TANAKA, K. & 6 others (1989). Molecular characterization of a functional cDNA for rat substance P receptor. *J. Biol. Chem.*, **264**, 17649.

YOUNG, D., WAITCHES, G., BIRCHMEIER, C., FASANO, O. & WIGLER, M. (1986). Isolation and characterization of a new cellular oncogene encoding a protein with multiple potential transmembrane domains. *Cell*, **45**, 711.

ZACHARY, I., SINNETT-SMITH, J.W. & ROZENGURT, E. (1986). Early events elicited by bombesin and structurally related peptides in quiescent Swiss 3T3 cells. I. Activation of protein kinase C and inhibition of epidermal growth factor binding. *J. Cell. Biol.*, **102**, 2211.

ZACHARY, I. & ROZENGURT, E. (1985). High-affinity receptors for peptides of the bombesin family in Swiss 3T3 cells. *Proc. Natl Acad. Sci. USA*, **82**, 7616.

ZACHARY, I. & ROZENGURT, E. (1987). Identification of a receptor for peptides of the bombesin family in Swiss 3T3 cells by affinity cross-linking. *J. Biol. Chem.*, **262**, 3947.

ZACHARY, I., WOLL, P.J. & ROZENGURT, E. (1987). A role for neuropeptides in the control of cell proliferation. *Dev. Biol.*, **124**, 295.

ZAGON, I.S. & McLAUGHLIN, P.J. (1987). Modulation of murine neuroblastoma in nude mice by opioid antagonists. *J. Natl Cancer Inst.*, **78**, 141.

ZURIER, R.B., KOZMA, M., SINNETT-SMITH, J. & ROZENGURT, E. (1988). Vasoactive intestinal peptide synergistically stimulates DNA synthesis in mouse 3T3 cells: role of cAMP, $Ca^{2+}$ and protein kinase C. *Exp. Cell Res.*, **176**, 155.